# Design of Light-Sensitive Triggers for Endothelial NO-Synthase Activation

**DOI:** 10.3390/antiox9020089

**Published:** 2020-01-21

**Authors:** Sébastien Dilly, Linda J. Roman, Nicolas Bogliotti, Juan Xie, Eric Deprez, Anny Slama-Schwok

**Affiliations:** 1 UMR CNRS 8200, Gustave Roussy Cancer Research Center, Université Paris-Saclay, 94607 Villejuif, France; Sebastien.Dilly@gmail.com; 2 Department of Biochemistry, University of Texas Health Science Center, San Antonio, TX 78229, USA; Linda.Roman@gmail.com; 3 PPSM, CNRS UMR8531, ENS Paris-Saclay, Université Paris-Saclay, IDA FR3242, F-94235 Cachan, France; Nicolas.Bogliotti@ens-paris-saclay.fr (N.B.); joanne.xie@ens-cachan.fr (J.X.); 4 LBPA, CNRS UMR8113, IDA FR3242, ENS Paris-Saclay, Université Paris-Saclay, F-94235 Cachan, France; deprez@lbpa.ens-cachan.fr; 5 Centre de Recherche Saint Antoine INSERM UMR S-938, Sorbonne Université, 75006 Paris, France

**Keywords:** endothelial NO-synthase, NADPH site, fluorescent trigger, docking, light-induced electron transfer

## Abstract

A specific light trigger for activating endothelial Nitric Oxide-Synthase (eNOS) in real time would be of unique value to decipher cellular events associated with eNOS activation or to generate on demand cytotoxic levels of NO at specific sites for cancer research. We previously developed novel tools called nanotriggers (NT), which recognized constitutive NO-synthase, eNOS or neuronal NOS (nNOS), mainly via their 2’ phosphate group which is also present in NADPH in its binding site. Laser excitation of NT1 bound to eNOS triggered recombinant NOS activity and released NO. We recently generated new NTs carrying a 2’ or 3’ carboxylate group or two 2’ and 3’ carboxylate moieties replacing the 2’ phosphate group of NADPH. Among these new NT, only the 3’ carboxylate derivative released NO from endothelial cells upon laser activation. Here, Molecular Dynamics (MD) simulations showed that the 3’ carboxylate NT formed a folded structure with a hydrophobic hub, inducing a good stacking on FAD that likely drove efficient activation of nNOS. This NT also carried an additional small charged group which increased binding to e/nNOS; fluorescence measurements determined a 20-fold improved affinity upon binding to nNOS as compared to NT1 affinity. To gain in specificity for eNOS, we augmented a previous NT with a “hook” targeting variable residues in the NADPH site of eNOS. We discuss the potential of exploiting the chemical diversity within the NADPH site of eNOS for reversal of endothelial dysfunction in cells and for controlled generation of cytotoxic NO-derived species in cancer tissues.

## 1. Introduction

Nitric oxide is a gaseous signaling mediator involved in physiological vasodilatation and neurotransmission, generated at low (20 to 100 nM) concentration by endothelial and neuronal nitric oxide synthases, eNOS and nNOS, respectively [1]. Low micromolar, cytotoxic NO levels generated by the inducible isoform iNOS contribute to the immune response [2,3] and fight against viral and bacterial pathogens. Dysregulation of NO biosynthesis leads to several pathologies, including septic shock, neurodegeneration, and inflammation [4,5,6]. Endothelial dysfunction is often linked to uncoupling of eNOS, which generates Reactive Oxygen Species (ROS) instead of NO under limited availability of cofactor tetrahydrobiopterin (H4B) [7]. eNOS activity is finely regulated by post-translational modifications and local micro-environment effects, i.e., Ca^2+^ flux, and by interactions with other proteins such as caveoline-1, which sequesters eNOS in an inactive state at the cell membrane [8]. To date, only eNOS transcription enhancer AVE9488 [9,10] and drugs indirectly promoting the phosphorylation of eNOS at S1177, including statins and ACE inhibitors, have been reported to enhance eNOS activity.

**Figure 1:**
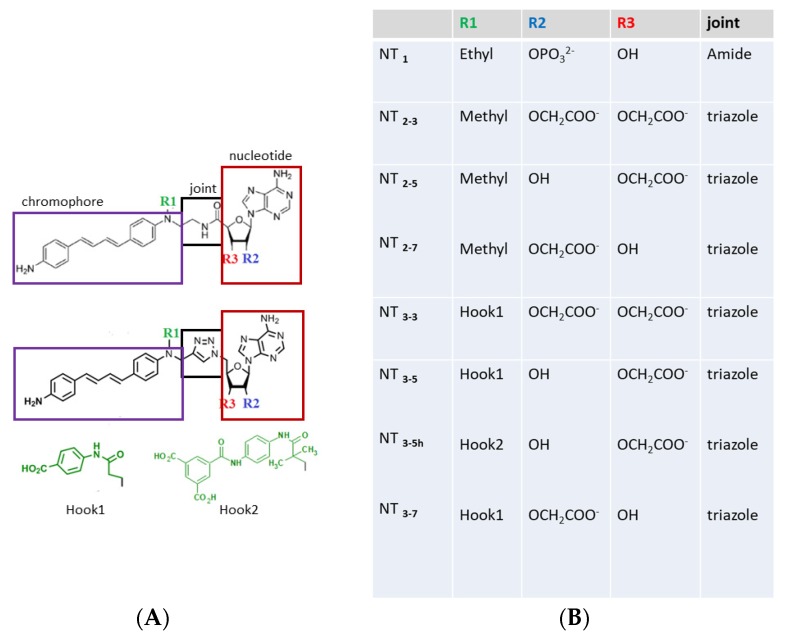
(**A**) Structures of the nanotrigger (NT) derivatives. In the prototype NT1 [16], the nucleotidic part boxed in red was kept identical to that of NADPH, as this moiety is responsible for interactions with conserved arginines found in the docking pocket in many NADPH- proteins [20,21]. The NADPH nicotinamide moiety was replaced by a photoactivable chromophore (purple box). An amide joint (black box) linked the nucleotidic and chromophore moieties. The R1 “hook” substituent of NT1 was an ethyl group. To further characterize how the charge carried by the ribose influenced binding to endothelial Nitric Oxide-Synthase (eNOS), the nucleotidic ribose was substituted by one or two carboxylate groups at either the 2’ or 3’ position or both, yielding NT2_7, NT2_5, and NT2_3, respectively [18,19]. In order to increase the affinity of the nanotrigger for e/nNOS, a charged R1 “hook1” was added, which targets additional charged residues of eNOS located within 7Ǻ. To increase both affinity and specificity, a second hook2 was designed to target variable residues of eNOS. (**B**) List of the compounds described or designed in this study.

Light-activatable tools to control eNOS activity that would in real time actitation and the ability to follow eNOS in precise cellular events, such as epigenetic modifications, would be of unique value. These tools would also be of great interest for time-resolved mechanistic studies, triggering initial catalytic events by laser activation and coupled with time-resolved X-ray crystallography. To date, most light-sensitive compounds are caged cofactors, such as caged NADPH, that would be activated upon light-induced uncaging or via light-induced isomerization [11,12]. Compounds of these types would be intrinsically limited by diffusion of the cofactor or the active moiety to its binding site, measured in milliseconds, too slow a timescale for many catalytic events, including eNOS catalysis [13,14]. To overcome the diffusion limitation, we have designed photoactivable NADPH derivatives targeting the NADPH binding site in the NOS reductase domain [15,16,17,18]. The first derivative, called nanotrigger NT1 (Figure 1), kept the nucleotidic part of NADPH, which was further linked to a conjugated, photoactivable chromophore substituted with two donor groups (Figure 1). Following photoactivation, the terminal amine group of excited NT1* injected electrons to the FAD acceptor of NOS, thereby initiating the electron flow. This ultrafast initiation and synchronization of NOS catalysis took place in 15–20 ps upon one and two-photon excitation, which led to light-induced NO formation [15,16]. NT1 bound to NOS with K_d_ = 7 ± 3 µM, but without isoform specificity for eNOS over nNOS [16]. Once bound, the fluorescence intensities of NT1 were enhanced, the absorption maxima were red-shifted while the emission maxima blue-shifted. A similar spectroscopic behavior was observed for the new NT derivatives bearing a carboxylate group instead of the phosphate group present in NT1 when bound to recombinant eNOS and in eNOS-containing cells [19]. Interestingly, enhanced two-photon emissions of several NT’s were observed in human umbilical vein endothelial cells (HUVECs) [15,19].

The main recognition elements of NADPH in its binding site in e/nNOS involve interactions of three conserved arginines with the 2’-phosphate group of NADPH [20]. This 2’ phosphate group was conserved in NT1, enabling the targeting of e/nNOS at their NADPH site in the reductase domain. In the second generation of NT, this phosphate group was substituted by either a 2’ or 3’ carboxylate group or both 2’ and 3’ carboxylate groups [18]. Interestingly only the 3’ carboxylate derivative was able to generate NO upon two-photon excitation. Here, we investigated the origin of these reactivity differences between the carboxylate-containing NTs using molecular modeling. These new NTs carried a “hook1” to target an additional arginine of e/nNOS and to compensate for the smaller charge of the 2’ or 3’ carboxylate of unsubstituted NTs as compared to 2’ phosphate of NT1. Fluorescence measurements consistently showed some affinity improvement by the hook1 implementation.

We then aimed to improve eNOS-specific binding to our NT by designing an eNOS-specific hook. Sequence alignments of NADPH binding sites within different proteins show a substantial variability within these sites, although their overall structure is conserved. Indeed, the nicotinamide moiety of NADPH can adopt various conformations in different NADP(H)-binding enzymes, showing a spatial separation between the catalytic subdomain involved in hydride transfer and the subdomain involved in molecular recognition via the 2’ phosphate group [20,21]. This suggested that there is a chemical space, in particular in the catalytic subdomain, to engineer enzyme specificity by taking advantage of the variability associated with each enzymatic catalysis. To achieve a selective activation of eNOS at the cellular and tissue levels by combining light activation—via electron injection of NT to eNOS after laser activation—with molecular targeting to eNOS, resulting in a photoactivatable sensor of eNOS, we used a fragment-based drug design approach to extend the nanotrigger with a fragment targeting variable and accessible residues of the NADPH site. These selected residues were addressed by a selective “hook” added to the NT. We discuss the potential of this strategy in reversal of endothelial dysfunction and associated epigenetic changes.

## 2. Materials and Methods

### 2.1. Computational Methods and Procedures

#### 2.1.1. Homology Modeling

The eNOS-human protein (UniProt identifier P29474, sequence 515-1178) and eNOS rat protein (UniProt identifier Q62600) were aligned on the nNOS-rat protein (UniProt identifier P29476, sequence 750:1413) and nNOS human protein (UniProt identifier P29475) using the ClustalW program. Based on the sequence alignment and using the coordinates of the monomeric form of the nNOS-rat (PDB id 1TLL [22]) as a template, a Swiss-PdbViewer project was made and submitted to the homology modeling website Swiss-Model Workspace. The missing loops were added automatically during the homology modeling. The resulting structure was refined by allowing the system to relax over molecular dynamics (MD) simulation. The model was further tested by Verify 3D and Ramachandran plots [23].

#### 2.1.2. Docking of the Nanotriggers into the Binding Site of eNOS

The nanotriggers were manually docked into the binding site of eNOS previously obtained by homology modeling with the nNOS-rat protein as a template [22]. The coordinates of the co-crystallized NADPH molecule were used to correctly superimpose the sugar-phosphate-adenine group of the nanotriggers and the NADPH. Moreover, the aniline group of the nanotriggers was positioned in order to mimic the stacking of the nicotinamide group with the isoalloxazine moiety of the FAD molecule in the active eNOS [23].

#### 2.1.3. Molecular Dynamics

For the MD simulations of the system formed by the dimeric form of the NOS reductase domain, the cofactors FAD and FMD, and NT3_3, NT3_5, NT3_7 and NT3_5h bound to eNOS, each complex was centered in a cubic box of edge length 80 Å; the box was subsequently filled with water using the Simple Point Charge (SPC) model and the system was made neutral using the Genion tool of the GROMACS package [24] (salt concentration of 0.15 mol.L^−1^). The resulting system then was subjected to energy minimization (200 steps of steepest descent followed 200 steps of conjugate gradient until the maximum force was smaller than 1000.0 kJ.mol^−1^ × nm^−1^) to remove steric clashes and to correct inappropriate geometry. Then, an equilibration step was applied to equilibrate the solvent and ions around the complex. Equilibration was carried out in two phases. The first was conducted in an NVT ensemble (N, constant number of particles, V, volume, and T, temperature) to reach a temperature of 310 °K using a velocity rescaling thermostat. The second phase was conducted in an NPT ensemble to equilibrate the system under 1 atm pressure using the v-rescale thermostat and the Parrinello-Rahman barostat. All equilibration phases were carried out during 2 ns. Upon completion of the equilibration step, the position restraints of the entire system were released for 20 ns production of molecular dynamic simulation (MDS) runs.

#### 2.1.4. Calculation of the Interaction Energies

For each complex, the interaction energies between the nanotriggers and eNOS complex were obtained by calculating the average value over the last 5 ns of the trajectory (i.e., once the complex was stabilized) and using the molecular mechanic potential energy component EMM of the g_mmpbsa tool of GROMACS (Royal Institute of Technology and Uppsala University, Uppsala, Sweden) [24]. The energy contribution of residues to the interactions with the 2’-phosphate or the 2’/3’ carboxylate groups of the nanotriggers was also evaluated using the same tool.

#### 2.1.5. Statistics

The distances (Ǻ) between the oxygen atom (OH) of Ser 941 and the central nitrogen atom (N sp2) of FAD, and, between the nitrogen atom (NH2) of NT compounds and the central nitrogen atom (N sp2) of FAD were measured along the 20 ns MD simulations using the gmx_distance tool of the GROMACS package version 2018 (Gromaacs is free tool developed at the Royal Institute of Technology and Uppsala University, Sweden to be downloaded at http://ftp.gromacs.org/pub/gromacs/gromacs-2018.tar.gz). Three replicates were carried out for each complex.

### 2.2. Experimental Procedures

The synthesis of the second generation of NTs (NT2_3, NT2_5, and NT2_7) and the third generation was previously reported [[18], NH Nguyen, PhD Thesis, 2015 ENS Cachan]. Synthetic protocols will be published elsewhere [25]. NTs were resuspended in DMSO to make 10 mg/mL stock solutions, which were diluted to 300 μM in buffer (50 mM Tris-HCl, pH 7.4, 100 mM NaCl). eNOS and nNOS proteins were recombinantly expressed and purified as described [26,27]. L-arginine, N-hydroxyarginine (NOHA), bovine calmodulin recombinant, CaCl_2_, (6R)-5,6,7,8-tetrahydrobiopterin (H4B) and NADPH were purchased from Sigma (Saint-Quentin-Fallavier, France).

The apparent binding affinities of the NTs to the neuronal (nNOS) and endothelial (eNOS) isoforms of nitric oxide synthase (NOS) were assessed by changes in NT intrinsic fluorescence using two types of titrations. In the first method, aliquots (1 μL) were added to 100 μL buffer containing NOS protein (5 μM), and fluorescence emission spectra were recorded from 430–700 nm (λexcitation = 420 nm; slit widths = 5 nm) on a Shimadzu RF-5301PC fluorimeter. Spectra of intrinsic protein fluorescence and unbound NT were subtracted from protein/NT spectra and the fluorescence at 460 nm was plotted against NT concentration. In the second method, we measured fluorescence spectra at a fixed concentration of the NT (1–2µM) with a Cary Eclipse spectrophotometer set at T = 20 °C, with excitation wavelength at 390 nm and emission wavelengths in the range 410–610 nm; the PM was usually set at 600 V with slits of 10 nm at both excitation and emission. The NT solutions with or without the nNOS substrate L-arginine or N-hydroxyarginine (NOHA) and with or without competitor NADPH were titrated by increasing concentrations of nNOS. Additional measurements of anisotropy of free NT and nNOS-bound NT were performed on the same apparatus as detailed in [28,29]. The steady-state fluorescence anisotropy was calculated using: *r* = (*I_VV_* − G × *I_VH_*)/(*I_VV_* + 2G × *I_VH_*) where *I_VV_* and *I_VH_* correspond to vertical and horizontal components, respectively, when the sample is excited with vertically polarized light, *G* is the *G*-factor accounting for the difference in the monochromator transmission between // and ⊥ polarized components (*G* = *I_HV_*/*I_HH_*).

Light irradiation of the NT -nNOS complexes in the presence of NOHA, H4B, and Ca^2+^/calmodulin was performed by a continuous irradiation with a Hg/Xe lamp (Hamamatsu, LC6 Lightningcure, 200 W) equipped with narrow-band interference filter to select 365 nm (Semrock FF01-370/36: center wavelength 370 nm, nominal bandwidth 40 nm) during one minute. The absorption changes before and after irradiation were measured on a Cary5000 spectrophotometer from Agilent Technologies (Les Ulys, France).

## 3. Results

### 3.1. Results

#### 3.1.1. Structures of the NT1 Nanotrigger Prototype and Design of Derivatives

NT1 is a photoactivable analogue of NADPH [15,16,17,18,23], designed to preserve the binding of the cofactor by the nucleotide moiety of the NADPH to its binding pocket within the NOS enzymes (red box in Figure 1). To introduce a photoactivable moiety in NT1, the flexible nicotinamide group of NADPH was replaced by a chromophore (purple box in Figure 1). This chromophore is composed of a dienic linker substituted by two phenyl moieties bearing a primary amine NH2 at one extremity and a tertiary amine at the other edge of the diene.

Our goals in this study were:

1. Test the effect of charge(s) carried by R2 or/and R3 on the ribose in the recognition of the conserved arginines of eNOS. Previous experimental data clearly emphasized the importance of electrostatic effects on their binding to eNOS by comparing the binding affinity of NT2_3, NT2_5 and NT2_7 with their diol derivative bearing no charge because both 2’ and 3’ positions were substituted by OH groups [18]. It was therefore logical to further explore these electrostatic effects by replacing the 2’ phosphate group by carboxylate group(s), one or two at the 2’ and 3’ positions of the sugar (R2 and R3 in Figure 1). To do so, we generated a compound containing two carboxylates, with a total charge of -2, instead of a phosphate group in NT1; we also generated compounds carrying a single carboxylate group either at the 2’ or 3’ position of the ribose with a charge of –1 (Figure 1); in the case of the carboxylate-bearing NT, a new triazole joint replaced the amide bond as it is considered to be its biostere and it can be formed by click chemistry [18].
2. Restore the loss of interaction energy inherent to the use of a single-charged carboxylate moiety (replacing the phosphate of NT1). To increase the affinity for e/nNOS, we re-engineered the previous NT [18] with an additional carboxylate substituted on a phenyl ring (R1 = hook 1) (Figure 1). Hook1 was expected to increase both the total charge of the compound and its hydrophobicity;
3. Improve affinity and specificity for the endothelial NOS, eNOS, with hook 2 (Figure 1) targeting eNOS-specific variable residues.

#### 3.1.2. Evaluation of the Substitution of the 2’ Phosphate Group by (a) Carboxylate Group(s) on the Nanotrigger Affinity for e/nNOS Determined by Fluorescence Measurements

**Figure 2:**
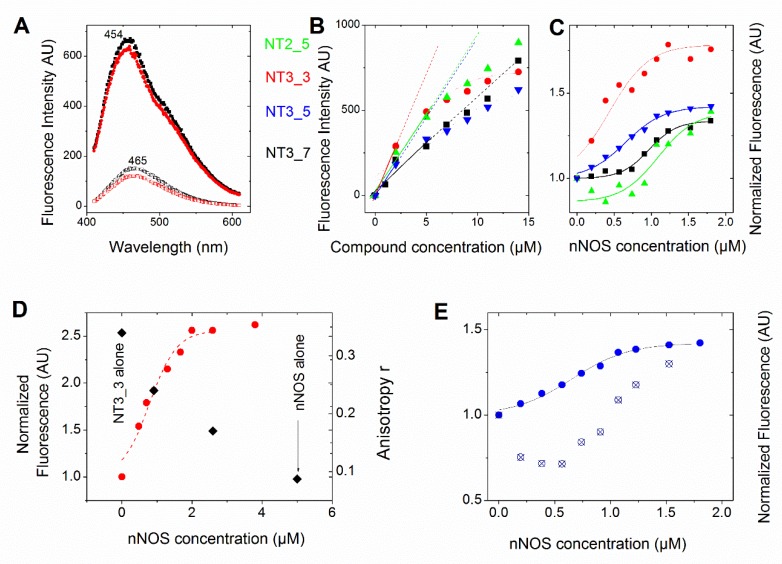
Comparison of the affinity of the NTs for NOS (see also Table 1). In all panels, we used the following color code: NT2_5 (green), NT3_3 (red), NT3_5 (blue), and NT3_7 (black) (**A**) Emission of free NT3_5 (2 µM) and NT3_7 (bottom spectra) showed an increased intensity and blue shift upon binding to nNOS (6 µM) (upper spectra). Fluorescence emission spectra were recorded from 420–680 nm (excitation λ = 390 nm; slits width = 10 nm). (**B**) Representative changes of fluorescence intensity of the protein-free NTs as a function of their concentration. The dotted lines are guidelines for the eye to highlight deviations from linearity even when corrected for inner filter effects. (**C**) Representative titration (one out of 2 at 1 and 2 µM NTs) of the NTs (1 µM) (using the above color code) with increasing concentrations of nNOS in buffer (10 mM Tris pH = 7.2, 100 mM NaCl). Each point is the mean of two measurements. Fluorescence emission spectra were recorded from 410–610 nm (excitation at 390 nm, slits width = 10 nm) and corrections were applied to substract intrinsic nNOS fluorescence. The fluorescence intensity at 460 nm was plotted against [nNOS] (usually up to 10 equivalents) and fitted by a single dose–response (Table 1). (**D**) 2 µM NT3_3 was titrated with nNOS in buffer containing 100 µM NOHA and the fluorescence changes at 460 nm were recorded (left scale, red circles). The fluorescence anisotropy at selected NT3_3/nNOS ratios is shown in the right scale as black squares. (**E**) Competition experiment of NADPH with NT3_5: 26 µM NADPH was added to 1 µM NT3_3 and nNOS at increasing concentrations as in C. The titration curve was shifted to higher nNOS concentrations, as expected. The Kd was extracted from the Cheng Prusoff equation Kd = IC50/(1 + [NADPH]/Km) using Km = 5.5 µM.

**Figure A1:**
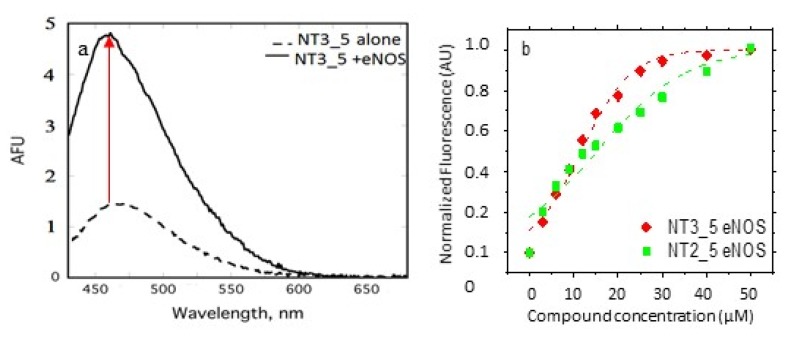
(**a**) Spectral changes of NT3_5 upon binding to eNOS. Fluorescence emission spectra were recorded from 430–680 nm (excitation = 420 nm; slit width = 5 nm) for NT3_5 (10 µM) alone and for eNOS (5 µM) + NT3_5 (10 µM). Spectrum of intrinsic protein fluorescence (due to flavins) was subtracted from protein+ NT spectrum. Fluorescence maxima were found at 470 nm and 450 nm for NT3_5 alone and eNOS + NT3_5, respectively, demonstrating an increase in fluorescence intensity and a blue shift upon binding to eNOS. (**b**) Titration of eNOS with NT2-5, NT3_5. Fluorescence emission spectra were recorded from 430–680 nm (excitation = 420 nm; slit width = 5 nm). Spectra of intrinsic protein fluorescence and unbound NT were subtracted from protein/NT spectra, and fluorescence at 460 nm was plotted. Representative data (out of four experiments in each NT) have been fitted by a single dose–response (NT2_5, NT3_5) (Table 1).

Figure 2A and Figure A1 show that the fluorescence intensities of NT3_3, NT3_7 and NT3_5 bound to e/nNOS are enhanced as compared to free NTs and presented a blue shift from 457, 462, and 465 nm to λ ≅ 454 nm. A similar behavior was observed for NT1 and the second generation of NTs [15,16,18]. This fluorescence increase allows for the determination of the apparent binding affinity of the various NTs for eNOS or nNOS. The binding curves of the monocarboxylated compounds to recombinant eNOS demonstrated a simple dose–response with apparent Kd ranging between 10 µM for NT3_5 and 16 µM for NT3_7 (Figure A1, Table 1), with corrections being made for contribution of (excess) free NT at a constant protein concentration.

**Scheme 1:**
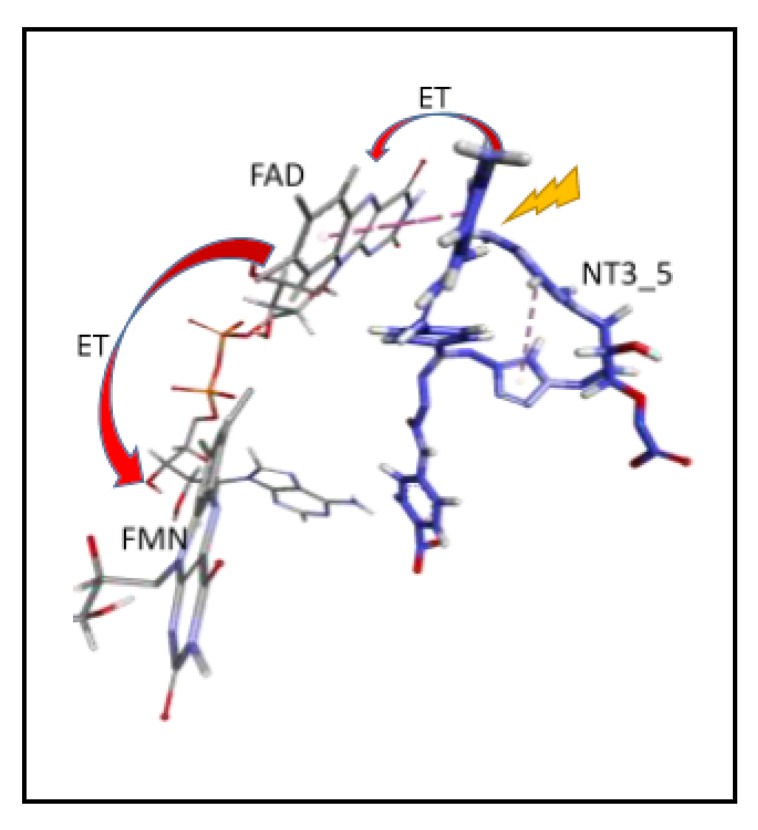
Proposed Electron Transfer Path From NT3_5* to FAD (flavin 5′-adenosine diphosphate), then Electron Transfer (ET) to FMN (Flavin mononucleotide) (, as in NADPH-Driven eNOS Catalysis [9,17].

**Figure A2:**
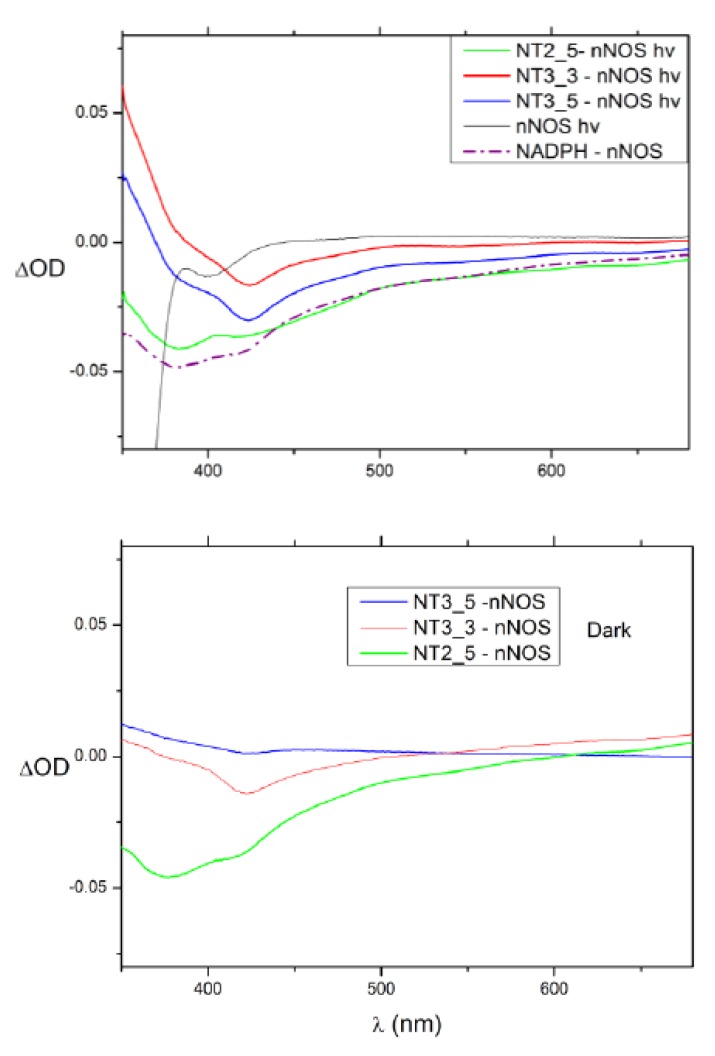
Upper panel: Spectral changes attesting to flavin reduction (minima around (380, 420, 460 nm) upon light irradiation at 365 nm: 2 µM bound NT2_5 and NT3_5 to 5 µM nNOS or NT-free nNOS as compared with absorption changes upon flavin reduction with NADPH; some spectral changes observed in the presence of NT3_3 or NT2_5 were also present without light irradiation; bottom panel shows the same conditions as above with no light. Spectral changes are observed with NT2_5 without light, suggesting it can reduce FAD in the ground state. Dark flavin reduction that is similar to light-induced reduction is also observed using NT3_3. This means that NT3_3 is not a light trigger and that its ground state redox potential is more reducing than NT3_5 is. Only NT3_5 reduced the flavin upon light irradiation. 100 mM NOHA, 100 mM Ca^2+^, 1 µg recombinant calmodulin, 20 mM Tris Buffer at pH = 7.8, 100 mM NaCl; representative data out of 2 experiments.

Table 1 also demonstrates the ability of the compounds to reduce nNOS flavins upon light excitation, a process initiated by injection of electrons from the NT* in its excited state to FAD (Scheme 1). NT1, NT2_5 and NT3_5 reduced FAD upon light excitation, with some ground state reduction only for NT2_5 (Table 1, Figure A2). In contrast, NT2_3 (and NT3_3) which bound somewhat tighter to nNOS than NT3_5 did could not reduce FAD nor generate NO. NT3_3 only showed ground state FAD reduction.

The above apparent affinity values were obtained assuming that the NTs did not self-associate and remained monomeric when they bound to e/nNOS in the concentration range used. To test this assumption, the fluorescence intensities at 460 nm of the free NTs in buffer were determined as a function of their concentration. As shown in Figure 2B, NTs fluorescence largely deviated from linearity above 5 µM, (even after correction for the inner filter effect, conditions in which the OD at excitation is usually higher than ≅ 0.1) in the order NT3_3 > NT3_5 > NT2_5 > NT3_7, showing that self-association of the NT took place at low micromolar concentrations.

Therefore, the titrations were performed at fixed and low concentrations of NTs (1–2 µM) with increasing nNOS concentrations. The spectra were corrected for the contribution of the nNOS protein alone. The experiments were performed in 10 mM buffer, 100 mM NaCl with or without substrate (i.e., L-arginine or N-hydroxyarginine, NOHA). The apparent affinities were lower than in the first set of experimental conditions, being in the range of 0.4–1.2µM using 1µM NTs (Figure 2C and Table 1).

To further test NT self-association in solution and how it was modified by binding to nNOS, we used fluorescence anisotropy. A freely rotating monomer during its emission lifetime as fluorescein exhibited a low anisotropy r = 0.005, but the anisotropy value of the nNOS-free NT3_3 at 5 µM was surprisingly larger r = 0.32 ± 0.02, indicating again that NT3_3 was not monomeric. Supported by fluorescence anisotropy, we could further validate that the self-association of NT in solution was disrupted upon binding to nNOS as the anisotropy of the NT-nNOS complexes decreased and tended to a similar r value to that of free nNOS (Figure 2D).

As we could not find suitable conditions to determine the NT self-association (equilibrium) constant, the apparent Kd values determined in the first method were not “real” values for the binding of monomeric NT to e/nNOS. In an attempt to minimize the self- association of the NTs, and be as close as possible to “real” values of a monomeric NT binding to nNOS, we used the low NT concentration of 1 µM still compatible with fluorescence intensities measurements (Table 1).

Finally, we performed competition experiments with NADPH as both NT and NADPH are competing for the same binding site. Binding of NT3_5 or NT3_3 was challenged with 26 µM NADPH. As expected, the added NADPH competed with NT3_5 for binding to the NADPH site of nNOS, showed by an increase of the apparent IC50 as shown in Figure 2E. Using the Cheng- Prusoff equation [30] and a Km value of NADPH for nNOS of 5.5 µM, Kd values of 0.18 ± 0.04 µM and 0.10 ± 0.03 µM for NT3_5 and NT3_3 binding to nNOS were calculated (Table 1).

Taken together, NT1 and NT3_5 similarly bound to the NADPH site of e/nNOS. This binding was characterized by a fluorescence enhancement of ca 1.5–2-fold (after correction for free nNOS fluorescence) and a blue shift of the fluorescence maximum. However, evidence for self-association was found for the second and third generation NTs as compared to NT1, which did not self-associate. Binding of the NTs to nNOS determined by direct titrations at the lowest possible concentrations of 1–2 µM exhibited smaller values of all NTs, i.e., 650 ± 150 nM for NT3_5, than that reported for NT1. Competition experiments showed that the binding constant of (monomeric) NT3_5 competing with NADPH for nNOS was Kd= 180 ± 40 nM. The mean value Kd = 360 ± 140 nM of NT3_5 for nNOS compared favorably with respect to Kd = 7 ± 3 µM determined for NT1. The order of Kd of the NTs for nNOS was: NT3_3 ≤ NT3_5 < NT3_7 < NT2_5. Additionally, the hook provided NT3_5 with improved affinity over that of NT2_5 for nNOS, which shows no specificity for nNOS over eNOS (Table 1).

NT1 [15] and NT2_5 [19] incorporated into HUVEC cells produced NO upon light activation. Based on our previous results with NT1 with its 2’ phosphate group, we anticipated better binding to NOS and more NO formed in HUVECs by NT2_7, with its 2’ carboxylate, over NT2_5, which has a 3’ carboxylate. This was not observed experimentally, with the same trend being followed by NT3_5 over NT3_7 among the third generation ([31]). These unexpected results based on the position of the charge on the ribose (2’ phosphate, 3’ carboxylate) prompted us to examine how these compounds bind to eNOS using docking and MD simulations, and to identify which binding differences could explain their differential ability to transfer electrons to FAD. Indeed, electron transfer will depend crucially upon the distance and orientation of the donor moiety (NH2 of NT chromophore) with respect to the primary electron acceptor FAD.

#### 3.1.3. Interactions of NTs with eNOS as Determined by MD Simulations; Impact of the Carboxylate Group(s) Versus the 2’ Phosphate Group

**Figure 3:**
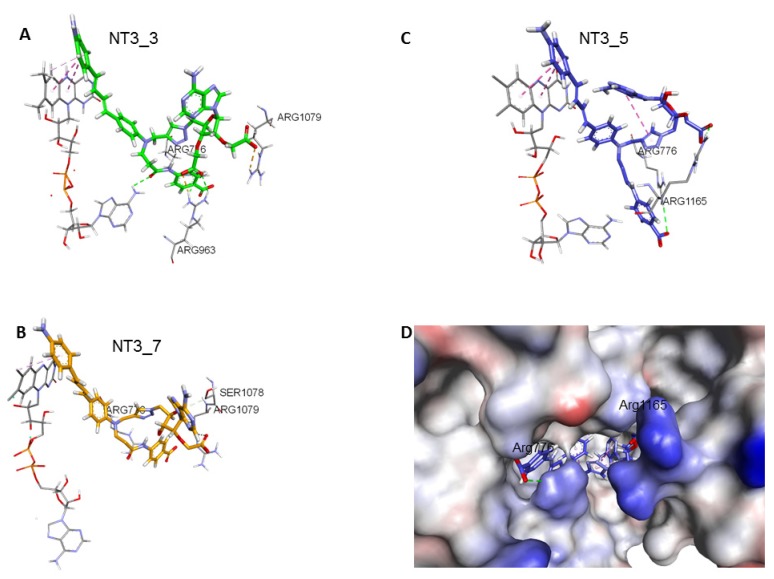
Comparison of NT3_5, NT3_7, NT3_3: effect of charge on key interactions between the nanotriggers bound to eNOS. Representative structures of the 3 replicas (**A**) NT3_3, (**B**) NT3_7, (**C**) NT3_5 for clarity, the view emphasizes only interactions of the carboxylate groups with argine residues of eNOS; the interactions between the NTs and eNOS are listed in Table 2 and their interaction energies summarized in Table 3; (**D**) solvent surface view of NT3_5 in the NADPH site; the view emphasizes R776 and R963 at the aperture of the site; FAD has been removed for clarity. Color code: blue positively interpolated charge, red negatively interpolated charge.

**Figure 4:**
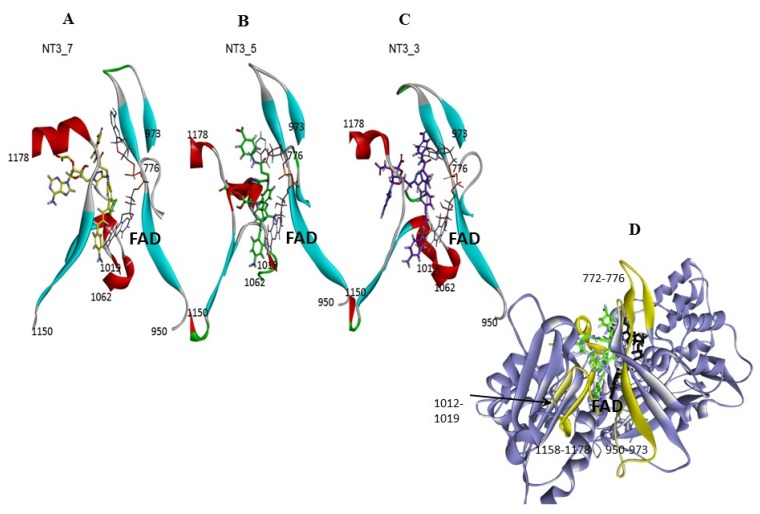
Gating elements of NT’s binding to the NADPH site of eNOS: the figure shows a simplified view of the NT3_7, 5, and 3, Panels A, B, C, respectively within their binding site. The gating elements are shown in yellow in Panel D (see also Figure A5 for a view with a 90° rotation). It is clearly seen that NT3_5 binding induced a fit of the protein due to its hydrophobic hub.

**Figure A3:**
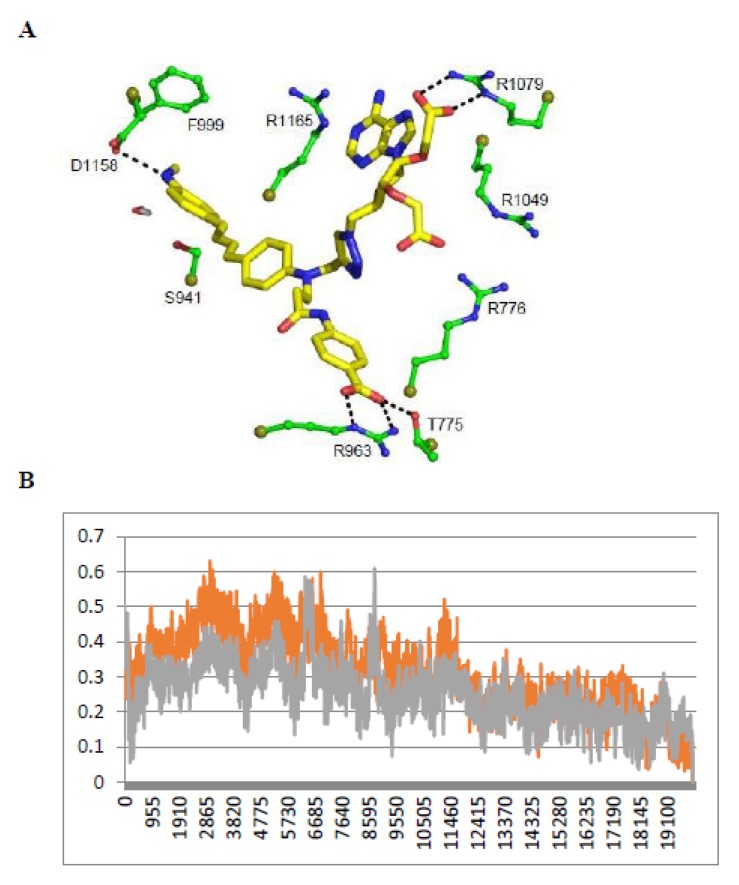
Stability of the 2 carboxylates of NT3_3: (**A**) Structure at 10 ns with only one carboxylate bound; (**B**) the fluctuations of the carboxylates: the 2’ (orange) and 3’ (grey) carboxylate of NT3_3 were quantified by the root mean square deviation (RMSD in Ǻ) during 20 ns of dynamics.

**Figure A4:**
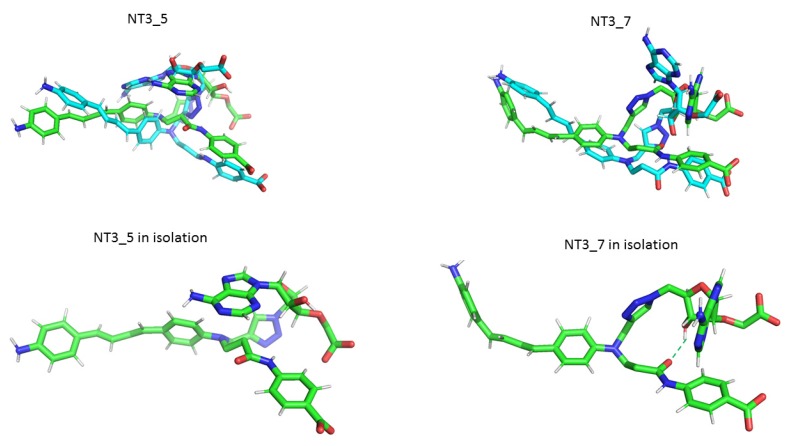
Comparison of NT3_5 and NT3_7 in isolation *(green)* and bound to eNOS *(cyan)*. The conformation of NT3_5 differed from that of NT3_7 by the relative planarity of its chromophore in the former compared to a largely non-planar chromophore of NT3_7. They differed also by the hydrophobic interactions between the triazole, the adenine, and the first phenyl ring of the chromophore of NT3_5, while stacking of the triazole with the adenine in NT3_7 was stabilized by an intramolecular H-bond between the amide of the hook and the OH of the ribose; the adenine helped to separate the charges of the hook and the nucleotide. Note the similarity between isolated and bound NT3_5 but the quite different conformations of NT3_7 when isolated and eNOS-bound, the protein limiting the bending of the chromophore with a significant modification of the adenine.

**Figure A5:**
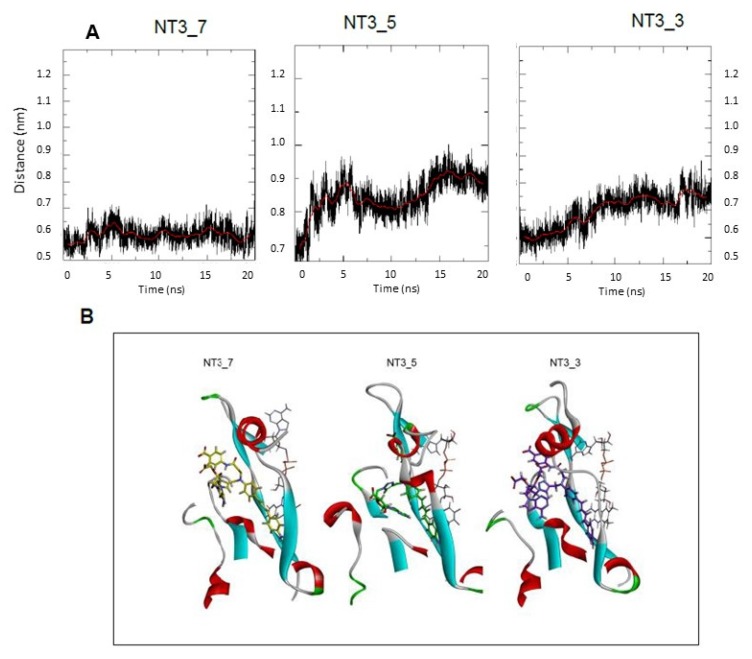
(**A**) Gating of NT 3_3, NT3_5 and NT3_7 binding to eNOS exemplified by the distance between A958 and T1016. One representative run of 20 ns out of three. Gating elements of NT’s binding to eNOS. (**B**) shows a simplified view of the NT3_7, 5, and 3 within their binding site. This view has a 90° rotation as compared to Figure 4 where the “gating” elements are shown in yellow.

- Stacking with FAD: As shown in Figure 3 and Table 2, the chromophores of the various NTs were close to the FAD isoalloxazine ring, with various degrees of stacking on FAD isoalloxazine rings. The terminal phenyl ring carrying the NH2 donor group of NT3_3 stacked on FAD (see the dotted pink lines emphasizing the hydrophobic contacts, Figure 3A). This was also the case for NT3_5 (see below). In contrast, NT3_7 did not stack well with FAD as its chromophore was relatively tilted to the side (Figure 3B).
- 2’ and/or 3’ carboxylate(s) interactions with eNOS: The 3’ carboxylate of NT3_5 bound to the conserved R1165. A view of NT3_5 fitted into its binding site is shown in Figure 3C,D, emphasizing the interactions with both R776 and R1165, which are residues located close to the protein- solvent interface, thus forming a charged “aperture of the site”. In NT3_7, the 2’ carboxylate moiety linked to the ribose ring bound to a conserved arginine of eNOS, R1079. The comparison of NT3_7 and NT3_5 interaction energies with eNOS showed a lower stability for the 2’ carboxylate in NT3_7 as compared to the 3’ carboxylate in NT3_5 (Table 3), in agreement with the experimental results. The total energy of interaction followed the electrostatic energies of the complexes (Table 3), namely NT3_3 > NT3_5 > NT3_7. Within error ranges, the interaction energies of NT1 and NT3_5 with eNOS were found to be comparable.
  As for NT3_3, the 2’ and 3’ carboxylate groups usually bound the conserved R1079 and the variable R963, respectively. To get more insight about a possible steric clash between the adjacent carboxylates on the ribose, hence a relative decreased stability, we computed the stabilities of both carboxylates along a number of MD runs (Figure A3). The 2’ carboxylate was found to be less stable than the 3’ carboxylate of NT3_3. Consequently, structures with only one carboxylate bound to eNOS co-existed (Figure A3) along with structures with both carboxylates bound (Figure 3A) during the three dynamics runs.
- Contribution of the hook R1: The 2’ phosphate of NT1 interacting with three conserved arginines R1049, R1079, and R1165, similar to the binding of the 2’ phosphate of NADPH [16,23]. As electrostatic interactions largely contributed to overall stability of the complexes, replacing the methyl groups of NT2_3, NT2_5, and NT2_7 with a charged hook R1 was expected to result in increased affinity of the compounds for NOS binding. The hook R1 formed an electrostatic interaction mainly with R776 in all three compounds; in NT3_3 and NT3_5; additional interactions of polar residues further stabilized hook1 (Table 2). Thus, hook1 was predicted to contribute to an increased stability of the protein-ligand complex as shown in Table 3, in agreement with experimental data (Table 1).
- Interactions induced by the triazole linker: We chose a triazole linker between the chromophore and the nucleotide (Figure 1) for its ease of generation by click chemistry [16,18]. The triazole was relatively rigid and participated in NT3_5 binding to NOS, as shown in Figure 3C. Extended stacking interactions were observed in NT3_5 between the triazole ring, the adenine ring, and P1014 that formed an “hydrophobic hub”. To adapt and contributing to this hydrophobic hub, one chromophore phenyl ring was somewhat twisted with respect to the first phenyl-NH2 moiety. This hub was further stabilized by I1018 and L1164, F1160 from the other side. In addition, R776 interacted with both the triazole and the benzoate ring and carboxylate group of hook1. In contrast, in NT3_7, the conformation of the triazole ribose was rather extended, the adenine being weakly stabilized by M1119. In NT3_3, the conformation of the triazole ribose was also rather extended. The adenine showed hydrophobic interactions with P1014 and L1164, as well as cation-π interaction with R1165.
- Protein-induced fit to NT3_5 binding conformation: Figure 3C shows that strong interactions between the triazole, the last phenyl ring of the chromophore, and the adenine ring contributed to stabilize NT3_5 in its binding pocket. This hydrophobic “hub”, only found in NT3_5, required the protein to adapt, with a β sheet (950–973) being pushed apart from a linker (1012–1019). The distance between A958 and T1016 thus increased to 9.5 Ǻ as compared to the same distance in NT3_3 and NT3_7 (6 and 8 Ǻ, respectively) (Figure 4 and Figure A4). The differences in interactions at the level of the ribose carboxylate and of the triazole–chromophore–adenine dictated the orientation of the chromophore and its ability to stack with the isoalloxazine ring of FAD. Table 2 and Figure 3 show that NT3_5 stacked better with the protein than NT3_7 did. It is likely that these structural features enhanced the ability of NT3_5 to transfer electrons upon light activation, as observed experimentally.
- Interestingly, the conformation of the NT 3_5 in isolation was similar to the eNOS-bound state, in contrast with NT 3_7 for which both conformations largely differed (Figure A4; see also Figure A5 for a view with a 90° rotation). A conformation close to the bound state of the nanotrigger in isolation reduces the energy necessary to unfold the nanotrigger and, also, the entropic barrier between the isolated and bound states, making NT3_5 a better ligand for binding to the protein than NT3_7.

#### 3.1.4. Toward an eNOS-Specific NT

**Figure 5:**
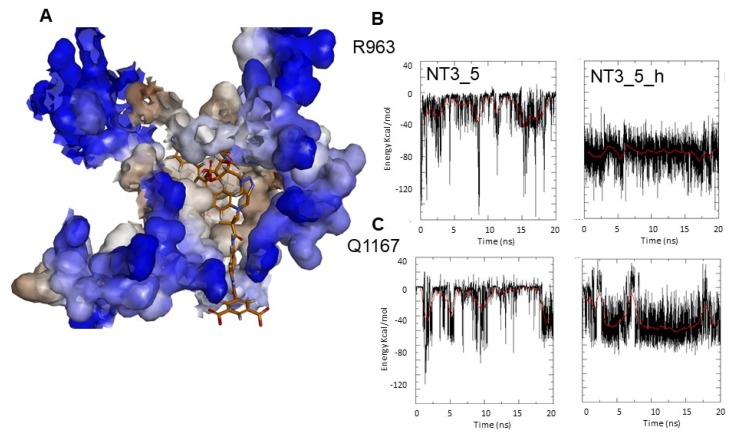
Targeting variable eNOS residues for selective binding. (**A**) Fit of NT3_5h into the NADPH site of eNOS represented in solvent surface view (using a probe radius of 1.5Ǻ) with the color code: hydrophylic blue and hydrophobic brown. The key interactions between the nanotrigger NT3_5h and eNOS are as follows: The NH2 terminal of NT3_5h formed an H-bond with D1158 and S941 stacked with the phenyl rings of the chromophore onto which adenine stacked. Extended interactions of P1014 with the chromophore, and of the 2’ hydroxyl and the 3’ carboxylate with R1165, stabilized this conformational folding. At the other edge, hook 2 recognized R776, R963, A961, Q1167. (**B,C**) Comparison of the Coulombic interaction energies of NT3_5 and NT3_5h for the variable residues R963 (B) and Q1167 (C) along one representative MD run out of three replicas. Note the stronger and more stable interactions in the presence of hook2 than hook1.

**Figure A6:**
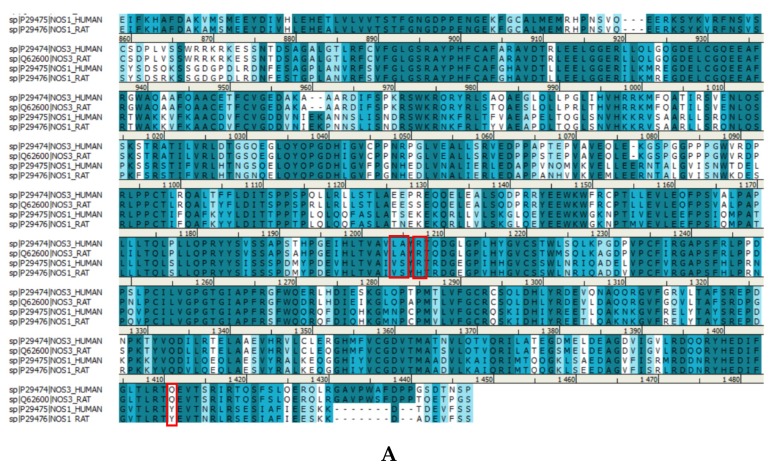

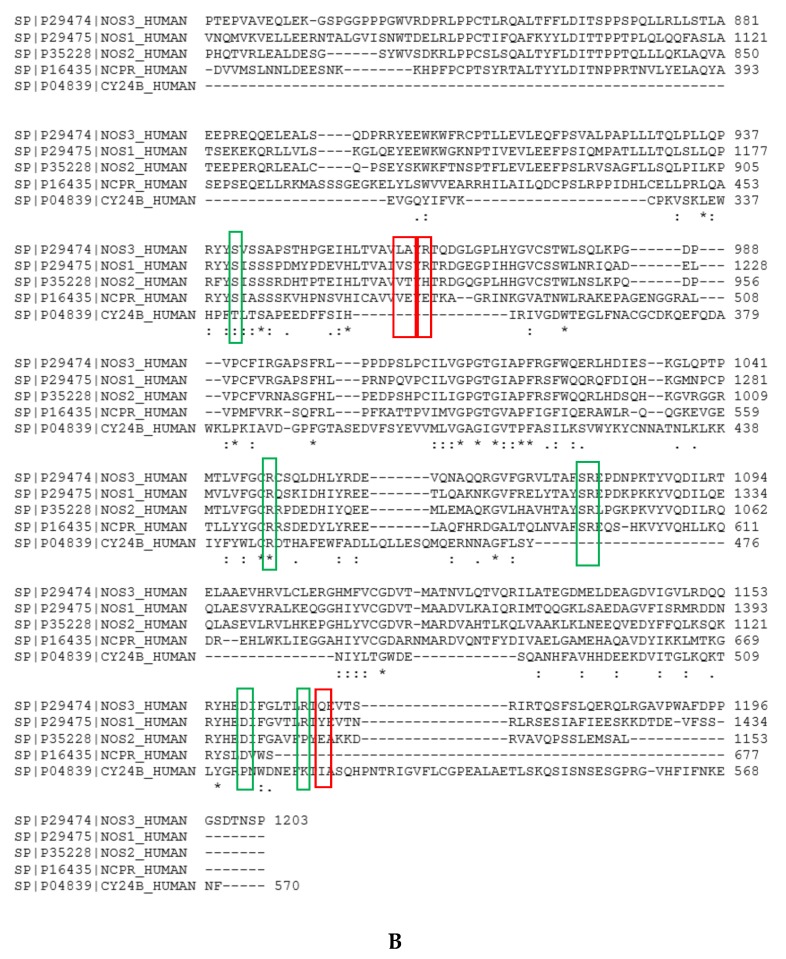
Targeting eNOS variable residues using multiple alignments. (**A**) The sequence alignment between the nNOS (NOS1) P29476 (750:1413) and eNOS (NOS3) P29474(515:1178) shows that 56.2% of residues are identical and 30.7% are similar, accounting for a 86.9% total homology. The color code is: deep blue: conserved residues, light blue or white: non conserved residues. The targeted residues, S1200, H1202, Y1426 for nNOS, and A961, R963 and Q1167 for eNOS, are highlighted in red. (**B**) Alignment of some human proteins containing NADPH sites: eNOS, nNOS, iNOS, P450 reductase and NOX2 (GP91phox). The conserved residues involved in the recognition of the charge (by 2’ phosphate of NADPH and of NT1, 2’carboxylate or 3’ carboxylate of NT3_7 or NT3_5) and of the catalytic S941 and D1158 are highlighted by a green box; R1049 is totally conserved among the five proteins, R776 (not shown), S941, D1158, S1078, and R1079 are conserved among the NOS and P450 reductase while R1165 is only shared by eNOS and nNOS. The targeted variable residues A961, R963 and Q1167 targeted by hook 2 are highlighted by a red box. We also found that L960, a variable residue unique to eNOS makes hydrophobic interactions with the chromophore of NT3_5h.

We selected NT3_5 as a starting point for implementing a specific moiety targeting eNOS. To do so, we designed a new hook, hook 2, to reach specific and variable residues of eNOS. We looked at residues spatially located close to the joint between the chromophore and the nucleotidic part. By sequence alignment, a number of residues were identified: A961, R963, and Q1167 of eNOS; the corresponding residues in nNOS were S1200, H1202, and Y1426 (Figure A6). Figure 5A shows that NT3_ 5h fitted well within the narrow NADPH site of eNOS, with hook2 turned at the charged aperture of this site (Figure 3D showing the NADPH site aperture as a charged surface). The presence of hook 2 slightly stretched the chromophore, which became more planar than that of NT3_5. A hydrophobic hub was still observed, with the stacking of the adenine on the chromophore and interacting with FAD isoalloxazine ring. Adenine was almost perpendicular to the triazole.

Figure 5B,C and Table 3 compared the electrostatic Coulombic interaction energies and the Lennard-Jones (LJ) energies corresponding to the van der Waals interactions of NT3_5 and NT3_5h with eNOS residues R963 and Q1167 along a 20 ns trajectory. The electrostatic interactions of R963 with NT3_5h and NT3_5 contributed –75 ± 20 and –30 ± 15 KJ/mol, respectively, while the LJ non-bonded interactions specifically stabilized NT3_5h by –20 ± 5 KJ/mol. The more discriminating Q1167 made more stable electrostatic and LJ interactions with NT3_5h (–50 ± 10 and –10 ± 4 KJ/mol, respectively) than with NT3_5 (–20 ± 15 and 0 KJ/mol, respectively). The contribution of A961 to NT3_5h and NT3_5 binding was small (data not shown). L960, a variable residue unique to eNOS, makes hydrophobic interactions with the chromophore of NT3_5h (Table 2). Taken together, hook2 contributed about 24% of NT3_5h total interaction energy with eNOS specific residues while hook1, as expected, only contributed 6.4% of NT3_5 total interaction energy for these targeted residues of eNOS (Table 3).

### 3.2. Discussion

**Figure A7:**
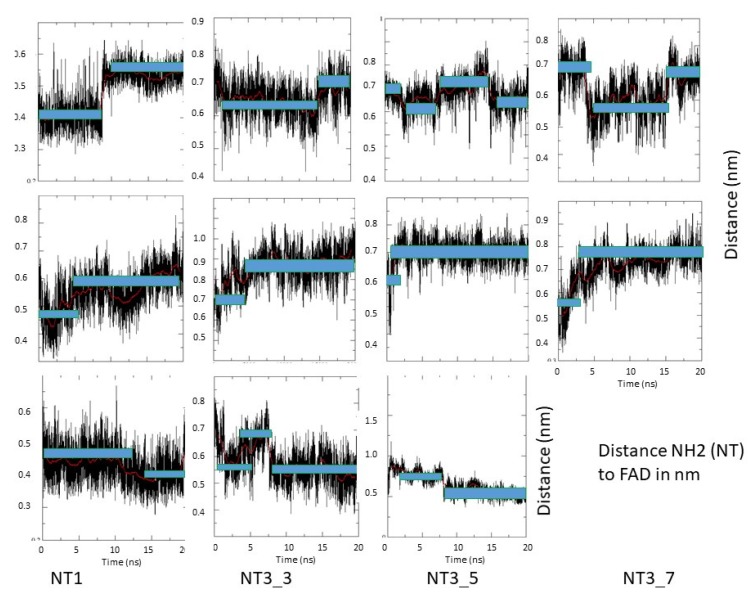
Fluctuations of the distance between the NH2 termini of NT1, NT3_3, NT3_5, and NT3_7 and FAD; the two main levels are highlighted by blue bars during one representative run of 20 ns out of three.

- Gate to the channel of the NADPH site: By inspecting Figure 3D, it is obvious that electrostatic interactions play an important role in the binding of any ligand to that site. The analysis of the dynamics (Figure 4 and Figure A4) shows that two of the three loops that were in the proximity of the NTs in eNOS (772–776 and 1012–1019) together with a β-sheet (950–978) and the C-terminal linker 1158-1178 formed a flexible aperture of the channel. These loops/linker movements guided the initial steps of the binding of the NTs and constituted a “gate keeper” to the channel. Along the entire path of NT binding, hydrophobic interactions guided the NTs to the conserved arginine region where the electrostatic interactions were the strongest. In addition to the electrostatic interactions with conserved arginines of eNOS, the chromophore and the adenine of NT3_5 contribute to binding by hydrophobic or polar contacts (A958, P1014, T1016, G1017, F1160, T1163, Table 2) and cation-π interactions (R776 and R963). The hook 1 in NT3_5 and hook2 in NT3_5h made additional electrostatic and hydrophobic interactions with one of the gate keeper loop (771–776), the β-sheet (950–978) and with C-terminal residues (Q1167) (Figure 5).
- Comparison between experimental and calculated interactions between NOS and the NTs: The interaction energies of NT1 and its monocarboxylate analogue at the 3’ position, NT3_5, were similar within the given error despite the fact that NT3_5 had a single charge at the level of the ribose and a charged hook1 contributing to binding while NT1 had two charges at the 2’ position of the ribose (Table 2). This is in agreement with the expected stabilization implemented by hook1, as observed experimentally (Table 1). Nevertheless, the interaction energies did not predict the experimental differences in Kd of about 20-fold given by the comparison of Kd = 360 ± 140 nM and 7 ± 3 µM for NT3_5 and NT1 binding to nNOS and eNOS, respectively [16]. Based on the anisotropy measurements (Figure 2D), we assume the following mechanism, whereby self-associated (NT)n needs to dissociate before binding to nNOS as a monomer. Such a self-association was not observed for NT1 [11,12] but was also observed for NT2_5 (Figure 2B). We hypothesize that these self-associated structures are at the origin of the cytotoxicities of the second generation of NT (as NT2_5) above 5 µM [14,15] in HUVEC cells, while NT1 was not toxic to HUVEC up to 20 µM.
- NT + nNT ⇋ (NT)n and NT + nNOS → NT-nNOS
- Electron transfer to e/nNOS (Scheme 1): NT1 and NT3_5 transferred electrons to the protein after light activation, which led to initiation of the electron flow in e/nNOS and experimental detection of NO [12,21]. In contrast, NT3_7, which bears a single carboxylate at the 2’ position, had a smaller interaction energy with eNOS than had NT3_5. NT3_7 chromophore did not stack well on the FAD isoalloxazine ring, suggesting a reduced electron transfer ability in the excited state, in agreement with its parent NT2_7 being unable to generate NO [18,19]. This is in agreement with previous studies on the effect of stacking interactions between flavin derivatives and aromatic hydride-donor model compounds [32]. We also computed the distance between the electron-donating NH2 terminal of the NTs and the electron acceptor FAD at the N5 position. It fluctuated between two levels in the various replica (Supplementary Figure A7): While in NT1, this distance fluctuated between 4.5 and 5.6 Ǻ, the levels ranged between 5.6 and 7.2 Ǻ for NT3_5, 6.2 and 7.5 and 5.7 and 7.7 Ǻ for NT3_3 and NT3_7. Although the distances were shorter for NT1 than NT3_5, favorable entropic (ring–ring) interactions in NT3_5 provided stabilization of the adenine by the first phenyl ring of the chromophore and further interactions with the triazole. Consequently, the two phenyl rings became twisted one with respect to the other. We cannot rule out that the excited state(s) may thus possess some character of a twisted intramolecular charge transfer state (TICT) with a high dipole moment, that would support the ability of NT3_5 or NT2_5 to transfer electrons to FAD. TICT was described for push–pull polyenes and merocyanines [33,34].
- Specificity for eNOS introduced by the hook 2. Here we target eNOS by exploiting sequence variations between the NADPH site of eNOS vs that of nNOS. Visual inspection of the sequence alignment (Figure A6) shows white or pale blue areas corresponding to variable residues, highlighting an available chemical space for implementing specificity for eNOS vs nNOS at this C-terminal domain. We choose to target A961, R963 and Q1167 of eNOS. By generating a more extended sequence alignment of human eNOS, nNOS, iNOS, P450 reductase, and NOX2, we can see that A961 with its nearby L960 and Q1167 were unique to eNOS and R963 was only shared with nNOS among nNOS, iNOS, P450red, NOX2. This suggested that by properly targeting variable residues, an expected selectivity should be achievable, and we used that information to design NT3_5h. Additionally, R1165, which only shared by eNOS and nNOS, was also recognized by the 3’ carboxylate of NT3_5 and NT3_5h. Indeed, the extended hook2 of NT3_5h contributed to multiple interactions with eNOS C-terminal and, overall, NT3_5h was well buried in its binding site (Figure 5). Its interactions with the variable residues were relatively stable over the trajectories. In contrast, hook1 of NT3_5 targeted a conserved arginine as R776 and its interactions with A961, R963 and Q1177 were relatively unstable (Figure 5B,C). The contribution of hook 2 to the NT3_5h interaction energy was at least 24%, based on its specific interaction energy with the variable residues A961, R963, and Q1167. Synthesis of this compound is ongoing for further experimental validation.

## 4. Conclusions

In conclusion, the modeling work offered some clues as to why a trigger bearing a 3’ carboxylate moiety is the only one among the second and third generation NTs that generates NO in endothelial cells upon light irradiation. We also describe the design of NT3_5h with characteristics of a eNOS-specific activator. Post-translational modifications [35] and epigenetic regulation [36] acutely impact eNOS activity, and dysregulation of these mechanisms compromise eNOS activity and foster the development of cardiovascular diseases. Post-translational modifications are often associated with changes in eNOS sub-cellular localization, which can be easily monitored and sensed by our family of NT’s light switches. Our switches do not require uncaging, nor do they require genetic modifications with respect to genetic encoding of light-sensitive proteins that activate signaling pathways in response to light. Our switches penetrate endothelial cells within minutes [18,19] and biphotonic excitation enables a great level of spatial control. Potential antitumoral application of the NT in the generation of cytotoxic NO generation addressed in a controlled way both in space and in time by appropriate biphotonic excitation could be envisioned.

## Figures and Tables

**Table 1:** Experimental Kd values obtained using the fluorescence changes of the NT compounds upon binding to eNOS or to nNOS (see Figure 2).

Compound	Apparent Kd eNOS (µM)	Hook1 Stabilization in eNOS	NO Formed upon Laser Excitation	Apparent Kd/Kd nNOS (µM)	Flavin Reduction hv	Hook1 Stabilization in nNOS
NT1	7 ± 3	NA	+	ND	+	NA
	[16]		[15,16]			
NT2_5	16 ± 3 ^a^	NA	+	19.5 + 1.1 ^a ^ [19]	+ ^e^	NA
	[19]		[19]	**1.3 ± 0.2 ^b^**		
				**4.0 ± 0.7 ^c^**		
NT3_5	10.7 ± 0.7 ^a^	×1.5	+ ^f^	11.0 ± 1.5 ^a^	+ ^e^	× 1.8
				**0.65 ± 0.15 ^b^**		
				**1.7 ± 0.5 ^c^**		
				**0.18 ± 0.04 ^d^**		
				**Mean ^(b,d)^: 0.36 ± 11**		**4–15**
NT2_7	24.1 ± 1.9 ^a^	NA	−	30 ± 4 ^a^	ND	NA
	[19]		[19]	[19]		
NT3_7	15.7 ± 0.6 ^a^	×1.5	− ^f^	11.5 ± 1,5 ^a^	ND	×2.6
				**0.90 ± 0.12 ^b^**		
NT2_3	14.7 ± 1.1 ^a^	NA	−	21.5 ± 0,7 ^a^	ND	NA
	[19]		[19]	[19]		
NT 3_3	5 ± 1 & 21 ± 0.2 ^a^	×2.9	− ^f^	5 ± 1 & 28 ± 2 ^a^	− ^e^	×4.3
	(bi-dose response)			**0.4 ± 0.1^b^**		
				**0.78 ± 0.15 ^c^**		
				**0.10 ± 0.03 ^d^**		
				**Mean ^(b,d)^: 0.25 ± 0.08**		

a: [eNOS] = 5–10 µM [NTs] = 0–50 µM (Figure A1); b: [NTs] = 1 or 2 µM, [nNOS] = 0–10 µM, 10 mM Tris pH = 7.2, 100 mM NaCl (Figure 2); c: [NTs] = 2 µM, [nNOS] = 0–10 µM, 10 mM Tris pH = 7.2, 100 mM NaCl, 100 mM NOHA; d: [NTs] = 1 µM, [NADPH] = 26 µM, [nNOS] = 0–5 µM, 10 mM Tris pH = 7.2, 100 mM NaCl; e: Figure A2; f: [25]; NA: non applicable; ND: not determined; +: NO formed under laser excitation; −: a lack of NO formed under laser excitation; the values in bold stands for real Kd while the values in plain character are apparent Kd.

**Table 2:** Main interactions of the NTs in their binding pocket in eNOS reductase domain.

NT Moities	NT1	NT3_3	NT3_5	NT3_7	NT3_5h
Phosphate or carboxylate	R1165, R1049, R1079	2’: R1165, S1078, R1079 3’: R963, L1164, R1165	3’: R1165, S1078, R1049	2’: R1079	3’: R1165, S1078 R1049, P1014 G1015
Hook, Ethyl ou Methyl	A958	R776, R963 Amide: Triazole	R776, R1165, V959, T1166, Q1167, Amide: T1163	R776, R1079	R776, T956 A961, R963 Q1167
Adénine	L1164, R1165 C1048, Y1087	P1014, M1119, L1164, R1165	A958, P1014, T1016, G1017, Triazole	T1087, M1119	R776, T956, A958 P1014, A1019, Triazole
Chromophore	Aniline: FAD, D1158, F1160, C1114,	Aniline: FAD, D1158, F1160, C1114	Aniline: FAD, D1158, F1160 A958, T1163, C1114	Aniline: FAD, D1158, C1114	FAD L960, C1114 D1158 (sometimes)
Chromophore	Planar	not planar	not planar	Planar,	Planar
Planarity/stacking to FAD	Stacking of the terminal aniline to FAD	Stacking of the terminal aniline to FAD	Stacking of the terminal aniline to FAD	bad stacking with FAD	Stacking of the terminal aniline to FAD
Distance (Ǻ) Ser941-FAD	4.7 ± 0.8	5.1 ± 0.4	5.1 ± 0.2	4.9 ± 0.5	5.5 ± 0.5
H-bond to FAD	S941, T956, V957, A958	A941, T956, V957, T956, T1016	T956, A958, T1016, G1017	S941, T956, V957, A958, T1016	S941, T956, V957, A958

**Table 3:** Interaction energies of eNOS with NT1, NT 3_3, 3_7, and 3_5, decomposed in Coulombic and Lennard-Jones (LJ) contributions to the total interaction energy determined by Gromacs. Note the decrease in electrostatic interaction energies for the 2’ carboxylate as compared to the 3’ carboxylate in NT3_7 and NT3_5. Note that hook1 added interaction energy in its binding to eNOS (total hook interaction energy −185 KJ/mol) but contributed only 6.4% (−40 KJ/mol) to the total interaction energy for binding specific variable residues. In contrast, most of the total interaction energy of hook 2 (−225 KJ/mol) with eNOS originated from specific interactions (−165 KJ/mol) with the targeted residues of eNOS. The values are mean values out of three replicas.

Compound	Total Interaction Energy (Kcal/mol)	Total Interaction Energy (KJ/mol)	Coulomb KJ/mol	LJ KJ/mol	Hook Total KJ/mol (% stabilization)	Hook Targeting eNOS Residues KJ/mol (%stabilization)
NT1	−155 ± 4	−647 ± 18	−343 ± 11	−304 ± 7	−19 ± 2	
NT3_3	−194 ± 3	−812 ± 12	−462 ± 3	−350 ± 9	−169 ±7 (21%)	
NT3_5	−150 ± 2	−627 ± 18	−277 ± 7	−350 ± 11	−185±15 (29.5%)	−40 ± 19 (6.4%)
NT3_7	−136 ± 2	−568 ± 10	−248 ± 7	−320 ± 3	−164 ± 18 (28.8%)	
NT3_5h	−163 ± 4	−681 ± 15	−279 ± 12	−402 ± 3	−225 ± 12 (33%)	−165 ± 35 (24.4%)
